# Kiperin Double-Hydrolyzed Collagen as a Potential Anti-Tumor Agent: Effects on HCT116 Colon Carcinoma Cells and Oxidative Stress Modulation

**DOI:** 10.3390/cimb47050364

**Published:** 2025-05-15

**Authors:** Lutfiye Karcioglu Batur, Cuneyd Yavas, Nezih Hekim

**Affiliations:** 1 Department of Molecular Biology and Genetics, Faculty of Engineering and Natural Sciences, Biruni University, Merkezefendi, 75 Sk No:1-13 M.G., 34015 Istanbul, Turkeynezihhekim@gmail.com (N.H.); 2 Biruni University Research Center (B@MER), Biruni University, 34015 Istanbul, Turkey

**Keywords:** double-hydrolyzed collagen, HCT116 colon carcinoma, cell viability, cell migration, oxidative stress, total antioxidant status

## Abstract

Double-hydrolyzed collagen, a key structural protein, has gained increasing attention for its role in cancer progression and its potential therapeutic applications. This study aims to investigate the effects of double-hydrolyzed collagen (Type I and III peptides) on HCT116 colon carcinoma cells and CCD-18Co fibroblasts as a normal control. Cells were treated with 0.5 µg/mL, 1 µg/mL, and 1.5 µg/mL of collagen peptide solution. HCT116 and CCD-18Co cells were cultured under standard conditions and treated with 1 µg/mL collagen. Cell viability (MTT assay), migration (scratch assay), oxidative stress (TAS/TOS kits), *TNF-α* expression (qRT-PCR), and tumor marker levels (CA19-9, CEA, CA72-4, and CYFRA 21-1; CLIA) were evaluated. Cell viability, proliferation, migration, oxidative stress, and tumor marker levels were assessed. Statistical analyses were performed to determine significance. Double-hydrolyzed collagen treatment significantly increased CCD-18Co fibroblast proliferation (*p* = 0.0143), while HCT116 cancer cell numbers significantly decreased (*p* = 0.0045). Migration of HCT116 cells was markedly reduced (*p* < 0.0001), whereas no significant effect was observed in CCD-18Co fibroblasts (*p* = 0.559). Oxidative stress analysis showed decreased total oxidative status (TOS) and increased total antioxidant status (TAS) in HCT116 cells (*p* = 0.0075 and *p* = 0.0095, respectively), with no significant changes in normal fibroblasts. Among tumor markers, CA19-9 levels were significantly reduced in HCT116 cells (*p* = 0.013), while CEA, CA72-4, and CYFRA 21-1 remained unchanged. *TNF-α* gene expression analysis confirmed the absence of inflammatory or adverse effects in normal fibroblasts. These findings suggest that double-hydrolyzed collagen selectively inhibits colon cancer cell proliferation and migration, modulates oxidative stress, and reduces CA19-9 levels while promoting fibroblast growth. The differential effects between cancerous and normal cells highlight collagen’s potential as a complementary therapeutic approach for colorectal cancer. Further research is needed to elucidate the underlying mechanisms and assess its clinical applicability. Double-hydrolyzed collagen appears to be a safe and beneficial dietary component with promising biological effects and therapeutic potential.

## 1. Introduction

Collagen, the most abundant protein in mammals, plays a critical role in maintaining the structural integrity of various tissues. Type I and III collagen, particularly abundant in connective tissues like tendons and skin, have recently gained attention not only for their benefits in skin and joint health, but also for their potential impact on cancer progression [1,2,3]. Recent studies suggest that collagen can modulate the tumor microenvironment, influencing cancer cell viability, metastasis, and immune responses [3,4,5]. Type I collagen, for instance, has been shown to provide structural support to the tumor matrix, promoting cancer cell survival and resistance to therapies by facilitating immune evasion [3,4]. Conversely, altering collagen production in cancer cells can enhance immune-mediated tumor suppression, as observed in various cancers, including colorectal carcinoma [6,7].

Colorectal carcinoma (CRC) is the third most common cancer worldwide and a leading cause of cancer-related deaths [8]. Its incidence increases with age, with lifestyle factors such as diet, physical inactivity, and obesity playing a significant role in its development [9]. Early symptoms include changes in bowel habits, rectal bleeding, and abdominal discomfort, though many cases are asymptomatic in early stages [10]. Treatment typically involves surgery, chemotherapy, and targeted therapies, depending on the stage of cancer [11]. Supplementation, particularly with bioactive compounds like collagen peptides, is of growing interest for its potential role in modulating tumor biology, improving patient outcomes, and reducing recurrence [12]. Incorporating supplements could complement conventional treatments by targeting oxidative stress and inflammation, which are key drivers in CRC progression [13].

Colorectal cancer (CRC) is among the most prevalent malignancies worldwide, with a high rate of morbidity and mortality. Various molecular and cellular factors contribute to CRC progression, including increased cell proliferation, enhanced migratory capacity, oxidative imbalance, and elevated levels of tumor markers. Tumor markers such as carcinoembryonic antigen (CEA) and carbohydrate antigen 19-9 (CA19-9) are widely utilized for CRC screening, diagnosis, and prognosis. While CEA is a well-established biomarker associated with tumor burden and metastasis, CA19-9 is particularly relevant in cases with mucinous histology and is often elevated in gastrointestinal cancers, including CRC. Other markers such as CA72-4 and CYFRA 21-1, although more commonly associated with gastric and lung cancers, respectively, have also been found to be elevated in subgroups of CRC patients and may provide additional information on disease heterogeneity or tumor aggressiveness [14,15].

Monitoring the levels of these tumor markers in in vitro models can offer valuable insights into the biological activity and potential therapeutic effects of novel agents. Moreover, evaluating oxidative stress markers such as TAS (total antioxidant status) and TOS (total oxidant status) helps to assess redox homeostasis, which is closely linked to inflammation-driven tumor progression. Therefore, a comprehensive assessment of viability, migration, oxidative stress, and tumor marker modulation provides a multifactorial perspective on how potential therapeutic compounds—such as bioactive peptides derived from double-hydrolyzed collagen—interact with malignant and normal cells [16].

Given the emerging interest in the biological activity of bioactive peptides, this study focuses on evaluating the potential anti-tumor effects of double-hydrolyzed collagen, which contains Type I and III collagen peptides derived from grass-fed, pasture-raised calves. The aim of the study is to investigate the in vitro effects of this compound on HCT116 colon carcinoma cells, specifically, assessing its influence on cell viability (via MTT assay), migration (scratch assay), oxidative stress (TAS and TOS assays), inflammatory response (*TNF-α* gene expression via qRT-PCR), and tumor marker levels (CA19-9, CEA, CA72-4, and CYFRA 21-1 using CLIA). Normal CCD-18Co colon fibroblasts were used as a control. Preliminary findings suggest that double-hydrolyzed collagen selectively reduces the proliferation and migration of cancer cells, modulates oxidative stress, and downregulates CA19-9 levels, without triggering inflammatory responses in normal fibroblasts. These results highlight the potential of this compound as a safe and biologically active complementary agent for colorectal cancer management.

## 2. Materials and Methods

### 2.1. Preparation and Characterization of Double-Hydrolyzed Collagen Peptides

The double-hydrolyzed collagen peptides used in this study were derived from bovine collagen, primarily sourced from hide and connective tissues. The collagen underwent controlled enzymatic hydrolysis using alcalase and neutral proteolytic enzymes under optimized conditions, specifically, at 45–60 °C and pH 6.5–8.5. Both single-step and sequential double hydrolysis protocols were employed. In the double-hydrolysis method, two complementary enzyme systems were applied in sequence to achieve a higher degree of hydrolysis and to obtain peptides with lower molecular weight. Throughout the process, peptide formation and molecular distribution were monitored. Upon completion of the hydrolysis, enzymatic activity was terminated, and the hydrolysate was subjected to centrifugation to remove insoluble residues. The resulting peptide-rich supernatant was then further purified using advanced fractionation techniques, including ultrafiltration and gel filtration chromatography. Peptide fractions were not only evaluated based on molecular weight, but also assessed for structural integrity, functional bioactivity, and physicochemical properties using spectroscopic and biochemical assays. The entire production process was optimized for reproducibility and scalability, demonstrating potential for industrial-level peptide generation. These procedures ensured that the collagen used in this study consisted of well-characterized, bioactive, and functional peptide forms.

Based on the official guidelines of the Turkish Ministry of Agriculture and Forestry, which recommend a maximum daily intake of 10 g of hydrolyzed collagen for dietary supplements [17], three physiologically relevant doses (0.5 µg/mL, 1 µg/mL, and 1.5 µg/mL) were selected for the experiments. These concentrations were proportionally adapted to the approximate number of cells (1 × 10^6^ cells/well) used in vitro and were chosen to ensure exposure levels that reflect realistic physiological conditions without inducing non-specific cytotoxicity.

### 2.2. Cell Culture

The study protocol was approved by Scientific Researches Ethical Committee of Biruni University on 12 December 2024, with a decree number of 2024-BIAEK/05-30. HCT116 colon carcinoma cells were obtained from ATCC (CCL-247) and CCD-18Co human colon fibroblasts cells, which were used as control group, were obtained from ATCC (CRL-1459).

CCD-18Co and HCT116 cells were cultured in Dulbecco’s Modified Eagle Medium (DMEM) supplemented with 10% fetal bovine serum (FBS) at 37 °C in a humidified incubator containing 5% CO_2_ (Kabin Incubator). The cells were maintained in these conditions throughout the experiments. The effective dose of collagen was determined as 1 µg/mL concentration among three doses tested (0.5 µg/mL, 1 µg/mL, and 1.5 µg/mL) by HCT116 cell counts measured three times using the Mindray BC-6800 (Shenzhen, China) cell counter. The two types of cell lines were divided into two experimental groups as follows: Control CCD-18Co (untreated) and Collagen-treated CCD-18Co (1 µg/mL concentration of collagen); Control HCT116 (untreated) and Collagen-treated HCT116 groups. Cells were incubated with the respective treatments for 48 h.

This study used HCT116 and CCD-18Co cells cultured under standard conditions. The effective collagen dose (1 µg/mL) was determined through triplicate measurements of cell counts for each tested concentration (0.5, 1, and 1.5 µg/mL). While biological replicates were not performed for initial cell culture experiments, all quantitative assessments were conducted with technical replicates to ensure reliability.

The test compound used in this study, collagen, was provided by Kiperin Pharmaceutical Food Industry and Trade Limited Company (Istanbul, Turkey). The product is composed of highly bioactive double-hydrolyzed collagen peptides (Type 1 and Type 3) derived from grass-fed, pasture-raised calf: 10 g.

### 2.3. Cell Scratch Assay

To evaluate cell migration, a cell scratch assay was performed. A confluent monolayer of cells was scratched with a sterile pipette tip, creating a uniform gap. Images of wound closure were taken at 6, 24, and 36 h using an inverted microscope at the same marked position. Wound healing was quantified by measuring the percentage of wound closure over time.

The cell scratch assay was performed once to evaluate migration, with three technical replicates (scratch wounds) per experimental group. Due to experimental constraints, independent biological repeats were not conducted for this assay.

### 2.4. Measurement of Oxidative Stress Levels and Anti-Oxidant Capacity

Cell lysates were prepared from HCT116 cells following standard protocols and stored at −80 °C until analysis. All reagents and samples were equilibrated to room temperature before use.

The total oxidant status (TOS) and total antioxidant status (TAS) levels in CCD-18Co and HCT116 cells were measured using commercial assay kits provided by Rel Assay Diagnostics (Mega Tıp San. Tic. Ltd. Sti., Gaziantep, Turkey). Both assays were performed according to the manufacturer’s protocol, utilizing a spectrophotometer (Epoch Microplate Spectrophotometer, BioTek Instruments, Winooski, VT, USA) to quantify the absorbance changes associated with the oxidative and antioxidative properties of the samples.

To measure TOS, the assay relied on the oxidation of ferrous ions to ferric ions by oxidant molecules in the sample. The ferric ions formed a colored complex with a chromogen in an acidic medium, and the color intensity was proportional to the total oxidant molecules present. For the assay, 45 µL of cell lysate, standard solution, or distilled water (used as a blank) was mixed with 300 µL of buffer solution (pH 1.75). The absorbance of the reaction mixture was first read at 530 nm after 30 s. Subsequently, 15 µL of ferrous ion solution was added to the mixture, and after incubation for 5 min at 37 °C or 10 min at room temperature, the absorbance was read again. The total oxidant status was calculated based on the change in absorbance and expressed as micromolar hydrogen peroxide equivalents per liter (µmol H_2_O_2_ Equiv./L).

For TAS measurements, antioxidants in the sample reduced dark blue-green ABTS radical cations to their colorless form. The assay used 18 µL of cell lysate, standard solution, or distilled water (blank), which was mixed with 300 µL of acetate buffer (pH 5.8). The initial absorbance of the reaction mixture was measured at 660 nm after 30 s. Subsequently, 45 µL of ABTS prochromogen solution was added, and after incubation for 5 min at 37 °C or 10 min at room temperature, the final absorbance was recorded. The antioxidant capacity was quantified based on the change in absorbance and expressed as Trolox equivalents per liter (mmol Trolox Equiv./L).

TOS and TAS levels were measured in cell lysates using commercial assay kits. Each sample was analyzed in triplicate to ensure precision, with two independently prepared lysates for biological validation. The TOS and TAS levels were calculated and analyzed statistically, with results expressed as mean ± standard deviation (SD).

### 2.5. RT-PCR for TNF-α Expression

Total RNA was extracted from cells, and the concentration was determined using a Qubit fluorometer. Subsequently, 500 ng of RNA was reverse transcribed into complementary DNA (cDNA) using the OneScript^®^ Plus cDNA Synthesis Kit (Applied Biological Materials Inc., Richmond, BC, Canada) according to the manufacturer’s instructions. The reverse transcription reaction was carried out with Moloney-Murine Leukemia Virus reverse transcriptase.

Quantitative real-time PCR (qRT-PCR) was performed using the BlasTaq™ 2X qPCR MasterMix (Applied Biological Materials Inc.) following the supplier’s protocol. Each reaction contained 2 µg of cDNA template, 1 µM of each primer, and the appropriate volume of MasterMix, adjusted to a final volume of 25 µL with nuclease-free water. The primers used were as follows: for *TNF-α*, forward 5′-CAGCCTCTTCTCCTTCCTGAT-3′ and reverse 5′-GCCAGAGGGCTGATTAGAGA-3′; for *GAPDH* (housekeeping gene), forward 5′-CCACCCATGGCAAATTCC-3′ and reverse 5′-TGGGATTTCCATTGATGACAAG-3′. Each reaction was performed in triplicate using *TNF-α* and *GAPDH* primers. While the experiment was performed in two biological replicates, technical replicates ensured measurement accuracy and relative gene expression was normalized to GAPDH using the ΔΔCt method, and results are presented as mean fold-change.

Amplification and detection were conducted on a Bio-Rad CFX96™ Real-Time PCR System (Bio-Rad, Hercules, CA, USA) under the following cycling conditions: initial denaturation at 95 °C for 3 min, followed by 40 cycles of denaturation at 95 °C for 15 s, annealing at 60 °C for 15 s, and extension at 72 °C for 15 s. Fluorescence data were collected at the end of each extension step. Relative gene expression levels were calculated using the ΔΔCt method, normalizing *TNF-α* expression to *GAPDH* as the internal control.

### 2.6. Analysis of Tumor Markers in HCT

The levels of CA19-9, CEA, CA72-4, and CYFRA 21-1 were quantified in HCT116 cells using chemiluminescent immunoassay (CLIA) systems as per the manufacturer’s instructions (Elecsys CA19-9, Elecsys CEA, and Elecsys CA72-4 kits (Roche Diagnostics GmbH, Mannheim, Germany), and the MAGLUMI CYFRA 21-1 kit (Snibe Co., Ltd., Shenzhen, China)). These assays utilize a sandwich CLIA technique to detect respective markers in cell lysates. Samples were incubated with monoclonal antibodies labeled with biotin or acridinium ester to form immune complexes, which were captured on streptavidin-coated magnetic beads or other solid phases. The luminescent signal was directly proportional to the marker concentration, measured in relative light units (RLUs). All reagents and analyzers were operated following the calibration and quality control guidelines recommended by the manufacturers. The assays were optimized for sensitivity and specificity, ensuring reliable quantification of tumor markers in vitro. Each marker was measured in triplicate (n = 3) from cell lysates, with consistent calibration and quality controls. Technical replicates were prioritized to maintain assay reproducibility, though biological replicates were not included.

### 2.7. Statistical Analysis

All statistical analyses were conducted using GraphPad Prism version 9.5.1 (GraphPad Software, San Diego, CA, USA).The distribution of the data was assessed for normality using the Kolmogorov–Smirnov test. For data that followed a normal distribution, comparisons between groups were made using the unpaired *t*-test. For non-normally distributed data, the Mann–Whitney U test was applied. A *p*-value of less than 0.05 was considered statistically significant.

## 3. Results

In this study, the impact of a highly bioactive double-hydrolyzed collagen was evaluated on the cell viability and migration, oxidative stress, antioxidant capacity, and tumor marker levels in HCT116 colon carcinoma cells and CCD-18Co fibroblasts.

To further investigate the dose-dependent effects of double-hydrolyzed collagen peptides, both HCT116 colon cancer cells and CCD-18Co normal colon fibroblast cells were treated with three different peptide volumes: 0.5 µL, 1 µL, and 1.5 µL. After 24 h of incubation, live cell counts were conducted.

In the HCT116 cell line, the number of viable cells was 2.829 × 10^9^ in the 0.5 µg/mL group, 1.605 × 10^9^ in the 1 µg/mL group, and 0.162 × 10^9^ in the 1.5 µg/mL group. The most pronounced anti-proliferative effect was observed at 1 µL, suggesting that this dosage may be within the optimal therapeutic range. However, a slight increase in viability at 1.5 µg/mL indicates a possible biphasic response at higher doses.

Additionally, for the CCD-18Co fibroblast cells, the number of viable cells was 1.921 × 10^9^, 3.63 × 10^9^, and 3.245 × 10^9^ at 0.5 µg/mL, 1 µg/mL, and 1.5 µg/mL, respectively. Across all concentrations tested, no significant cytotoxic effect was observed in the normal fibroblasts, indicating a selective anti-proliferative effect of the peptides toward malignant cells.

### 3.1. Cell Viability and Proliferation

**Figure 1:**
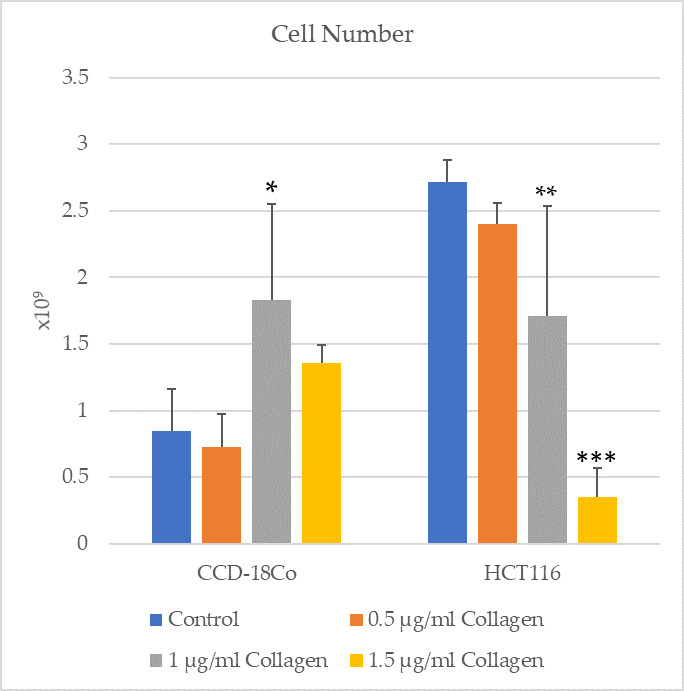
Dose-dependent cell numbers of CCD-18Co and HCT116 groups. * *p* < 0.05, ** *p* < 0.01, *** *p* < 0.001 vs. control group. Error bars indicate the standard deviation, reflecting the variability within experimental replicates (*n* = 3).

The cell numbers of CCD-18Co and HCT116 groups were assessed in control conditions and after treatment with 1 µg/mL collagen (Figure 1). The results indicate a significant increase in cell number of the CCD-18Co group treated with collagen compared to the control group (*p* = 0.0143). Similarly, a significant reduction in cell number was observed in the HCT116 group treated with collagen compared to the control group (*p* = 0.0045). These findings suggest that collagen treatment has distinct effects on normal fibroblast cells (CCD-18Co) and cancerous epithelial cells (HCT116), promoting proliferation in fibroblasts while inhibiting cell growth in cancer cells.

### 3.2. Cell Migration

**Figure 2:**
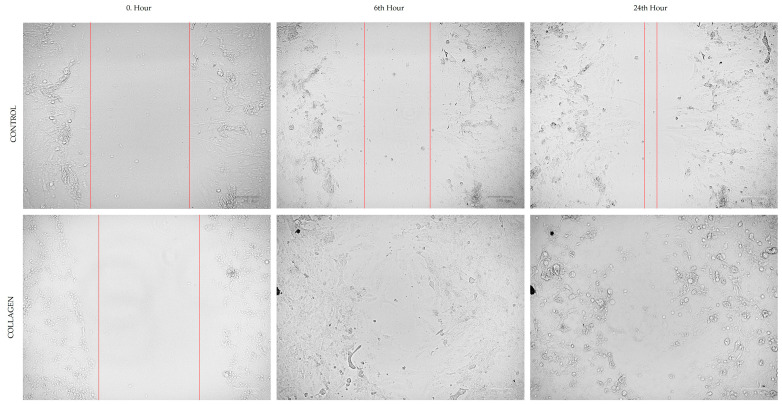
The images of cell migration assay of CCD-18Co cells. Red lines indicate the cell boundaries at each time point, reflecting the progression of migration and proliferation. Image taken at 700× magnification.

**Figure 3:**
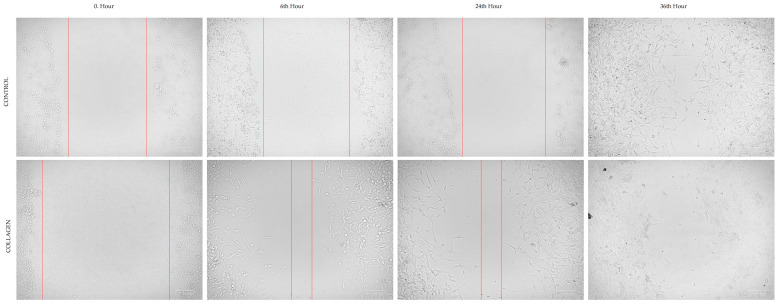
The images of cell migration assay of HCT116 cells. Red lines indicate the cell boundaries at each time point, reflecting the progression of migration and proliferation. Image taken at 700× magnification.

The optimum migration distance was measured at 24 h, which was selected as an effective time point to measure the cell migration for both CCD-18Co and HCT116 cells (Figure 2 and Figure 3). Table 1 presents the migration rates (%) of CCD-18Co and HCT116 cells in the cell scratch assay under control conditions and after treatment with 1 µg/mL collagen. For CCD-18Co cells, the migration rate showed a slight decrease in the collagen-treated group (45.81 ± 5.51%) compared to the control group (48.54 ± 9.66%), although this difference was not statistically significant (*p* = 0.559). In contrast, the migration rate of HCT116 cells was significantly reduced in the collagen-treated group (56.86 ± 6.89%) compared to the control group (81.31 ± 2.65%, *p* < 0.0001). These results suggest that collagen treatment specifically inhibits the migration of HCT116 colorectal cancer cells, while having no significant impact on the migration of CCD-18Co fibroblasts.

### 3.3. Oxidative and Antioxidant Status

Table 2 shows the TOS and TAS levels in CCD-18Co and HCT116 cell groups treated with 1 µg/mL collagen compared to control conditions. In CCD-18Co cells, TOS levels were higher in the collagen-treated group (13.365 ± 10.976 µmol H_2_O_2_ Equiv./L) compared to the control group (7.273 ± 4.273 µmol H_2_O_2_ Equiv./L), though this difference was not statistically significant (*p* = 0.686). TAS levels in CCD-18Co cells decreased in the collagen-treated group (0.040 ± 0.017 mmol Trolox Equiv./L) compared to the control (0.305 ± 0.207 mmol Trolox Equiv./L), also without statistical significance (*p* = 0.400).

In contrast, significant differences were observed in HCT116 cells. TOS levels were significantly lower in the collagen-treated group (7.69 ± 1.54 µmol H_2_O_2_ Equiv./L) compared to the control group (14.29 ± 0.88 µmol H_2_O_2_ Equiv./L, *p* = 0.0075). TAS levels in HCT116 cells were significantly higher in the collagen-treated group (0.103 ± 0.077 mmol Trolox Equiv./L) compared to the control group (0.003 ± 0.006 mmol Trolox Equiv./L, *p* = 0.0095). These findings suggest that collagen treatment reduces oxidative stress and enhances antioxidant capacity in HCT116 cells, while its effects on CCD-18Co cells are less pronounced.

### 3.4. TNF-α Expression Level

**Figure 4:**
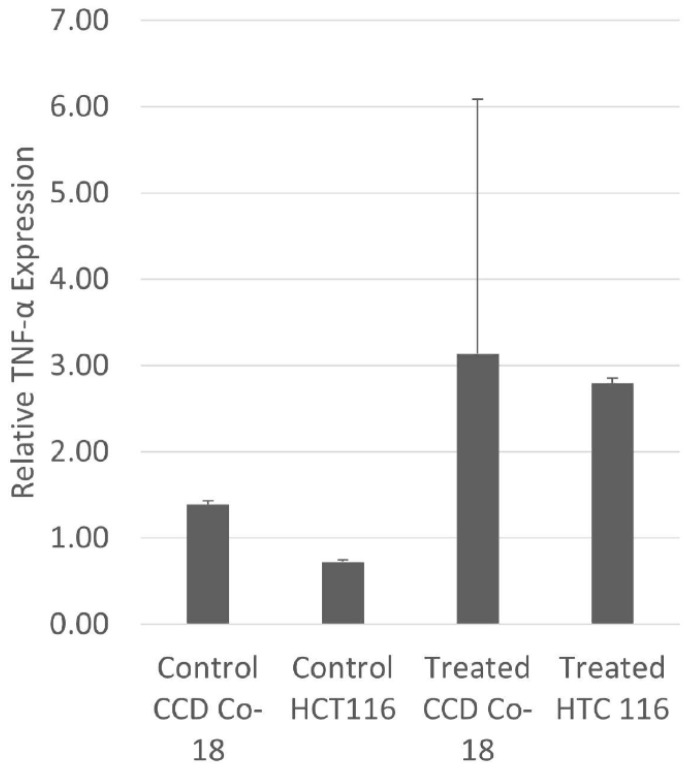
*TNF-α* expression levels in HCT116 and CCD-18Co cells following collagen treatment. Quantitative real-time PCR analysis of *TNF-α* mRNA expression in HCT116 colon carcinoma cells and CCD-18Co normal colon cells after treatment with collagen. Data are presented as mean ± standard deviation (SD) from three independent experiments. Statistical analysis using Student’s *t*-test showed no significant difference between treated and control groups in both cell lines (HCT116: *p* = 0.3333; CCD-18Co: *p* = 0.5547).

The results of RT-PCR for *TNF-α* expression indicated no statistically significant differences between the treated and control groups (*p* = 0.5547 for CCD-18Co and 0.3333 for HCT116). These findings suggest that collagen treatment does not significantly alter *TNF-α* expression in these cell lines under the conditions tested (Figure 4).

### 3.5. Cancer Marker Levels

Table 3 presents the levels of tumor markers CA19-9, CEA, CA72-4, and CYFRA 21-1 in HCT116 cell groups treated with 1 µg/mL collagen compared to control conditions. The CA19-9 levels significantly decreased in the collagen-treated group (1.04 ± 0.21) compared to the control group (4.61 ± 0.69, *p* = 0.013). CEA levels were below the detection limit (<0.2) in both control and collagen-treated groups, showing no change with treatment. Similarly, no significant differences were observed in the levels of CA72-4 and CYFRA 21-1. CA72-4 levels were slightly lower in the collagen-treated group (0.84 ± 0.53) compared to the control group (1.03 ± 0.46, *p* = 0.593), whereas CYFRA 21-1 levels were similar between the collagen-treated (2.73 ± 0.32) and control groups (2.71 ± 0.64, *p* = 0.857). These findings suggest that collagen treatment specifically reduces CA19-9 levels in HCT116 cells, while other tumor marker levels remain unaffected.

## 4. Discussion

The present study investigated the effects of 1 µg/mL collagen treatment on CCD-18Co fibroblasts and HCT116 colorectal cancer cells, focusing on cell proliferation, cell migration, oxidative stress markers, and tumor marker levels. The novel formulation of Kiperin provides bioactive collagen peptides and additional natural compounds, aimed at supporting various physiological processes related to tissue repair and inflammation modulation. The findings revealed that collagen treatment increased cell numbers in CCD-18Co cells while decreasing them in HCT116 cells. The collagen treatment decreased the migration rates of HCT116 colorectal cancer but not those of CCD-18Co fibroblasts. Additionally, collagen treatment led to a reduction in TOS and an increase in TAS in HCT116 cells, with no significant changes observed in CCD-18Co cells. Furthermore, collagen treatment significantly decreased CA19-9 levels in HCT116 cells, whereas other tumor markers remained unaffected. In our study, treatment with collagen did not induce any pro-inflammatory response in healthy cells, as evidenced by unaltered *TNF-α* gene expression levels, indicating the absence of inflammation.

The observed increase in CCD-18Co cell proliferation upon collagen treatment aligns with the existing literature indicating that collagen can promote fibroblast activation and proliferation [18,19]. Hydrolyzed collagens or collagen peptides with molecular sizes ranging from <3 to 3000 KDa promote the stimulation of fibroblasts in human tissues [18]. For instance, a study demonstrated that TL1A, a cytokine involved in fibrosis, enhances fibroblast proliferation and collagen deposition in CCD-18Co cells through the TGF-β1/Smad3 signaling pathway [19]. This suggests that collagen may similarly stimulate fibroblast proliferation via activation of pro-fibrotic signaling pathways.

Conversely, the reduction in HCT116 cell numbers following collagen treatment may be attributed to the modulation of the tumor microenvironment by collagen, potentially inhibiting cancer cell proliferation. Co-culture studies have shown that interactions between fibroblasts and colorectal cancer cells can influence cancer cell behavior, with fibroblasts either promoting or inhibiting proliferation depending on the context [20]. The decrease in HCT116 cell numbers suggests that collagen may alter the microenvironment in a manner that suppresses cancer cell growth. Although CCD-18Co normal human colon fibroblasts were used to assess the effects of double-hydrolyzed collagen on non-cancerous cells, future studies are needed to evaluate its impact on additional normal cell types, such as normal colon epithelial cells, to comprehensively understand the selectivity and safety profile of collagen peptides. This limitation has been acknowledged and highlights the importance of expanding the experimental models in future research.

The results of the cell scratch assay indicated that collagen treatment did not significantly affect the migration of CCD-18Co cells, but notably inhibited the migration of HCT116 cells. These findings align with the existing literature highlighting collagen’s dual role in cancer progression. Collagen, a major component of the extracellular matrix (ECM), can influence tumor cell behavior through interactions with integrins and other receptors, affecting cell adhesion and migration [12]. In colorectal cancer, collagen remodeling has been shown to impact cancer cell migration and invasion [21]. The observed inhibition of HCT116 cell migration upon collagen treatment may be attributed to alterations in the ECM that hinder cancer cell motility. Conversely, the lack of significant effect on CCD-18Co fibroblast migration suggests that collagen’s influence may be context-dependent, varying between normal and cancerous cells. Studies have shown that collagen can promote fibroblast proliferation and migration, which are essential for wound healing and tissue repair [22]. However, in the present study, collagen treatment did not significantly alter fibroblast migration, indicating that the response to collagen may differ based on cell type and environmental factors. Overall, these findings emphasize the importance of extracellular matrix composition and cell-type specificity in regulating cellular responses to collagen [23].

The significant reduction in TOS and increase in TAS observed in HCT116 cells upon collagen treatment indicate a shift towards a more antioxidative state. This finding is consistent with studies reporting that certain treatments can modulate oxidative stress in cancer cells, thereby affecting their survival and proliferation [24]. The decrease in oxidative stress markers suggests that collagen may exert protective effects against oxidative damage in HCT116 cells.

Although several studies have reported that certain forms of collagen, particularly matrix-associated fibrillar collagen, can support tumor growth and metastasis through interactions with integrin receptors and the tumor microenvironment, the findings of the present study highlight a contrasting effect [25]. The collagen used in our study is a double-hydrolyzed, low-molecular-weight peptide form derived from grass-fed calves, which lacks the structural integrity and ECM-binding properties of native collagen fibers. Unlike native collagen, which can promote tumor progression by activating signaling pathways such as PI3K/Akt or FAK through mechanical and adhesive cues, hydrolyzed collagen peptides may act through distinct biochemical mechanisms, including modulation of oxidative stress, suppression of inflammation, and alteration of gene expression related to cell proliferation and migration.

This distinction underscores the importance of collagen form and context. Our results suggest that hydrolyzed collagen peptides do not mimic the tumor-supportive characteristics of intact collagen in the extracellular matrix, but rather exert selective anti-proliferative and anti-migratory effects on colorectal cancer cells [26]. These findings are in line with previous studies that reported tumor-inhibitory activities of bioactive peptides, including marine- and bovine-derived collagen fragments.

In our study, collagen treatment does not significantly alter *TNF-α* expression in these cell lines under the conditions tested. *TNF-α* is a pro-inflammatory cytokine involved in various cellular processes, including inflammation, apoptosis, and cell proliferation. Elevated levels of *TNF-α* have been associated with cancer progression and metastasis, particularly in colorectal cancer. Studies have shown that *TNF-α* can promote tumor proliferation, migration, invasion, and angiogenesis [27]. Collagen, as a major component of the extracellular matrix, plays a crucial role in the tumor microenvironment and can influence tumor cell behavior through interactions with various receptors and signaling pathways [12]. However, the specific effects of collagen peptides on *TNF-α* expression in colon carcinoma cells are not well-documented. Some studies have indicated that collagen peptides can ameliorate intestinal epithelial barrier dysfunction by enhancing tight junctions, suggesting potential anti-inflammatory properties [28]. Our findings indicate that collagen does not significantly impact *TNF-α* expression in HCT116 colon carcinoma cells or CCD-18Co normal colon cells. This suggests that collagen’s effects on these cell lines may not involve modulation of *TNF-α* levels. Further research is needed to elucidate the mechanisms by which collagen interacts with cellular pathways and to determine its potential therapeutic applications in cancer treatment (Figure 4). The fact that collagen administration did not significantly alter TNF-α expression suggests that collagen does not directly affect cellular inflammatory responses. However, these results raise the possibility that collagen may be effective on other inflammatory mediators or alternative signaling pathways other than TNF-α. The indirect effects of collagen on the immune response can be further elucidated by investigating whether there is a change in the expression levels of different inflammatory regulators such as NF-κB, IL-6, or IL-10 [29] using the quantitative real-time polymerase chain reaction (qRT-PCR) restriction fragment length polymorphism (RFLP) method for monitoring highly conserved transgene expression during gene therapy. For translational research, it should also be considered that the effects of collagen administration may be mediated not only at the mRNA level, but also through post-translational modifications, protein stability, or secretion mechanisms [30]. Therefore, in future studies, proteomic analyses or secretome profiling can be performed to evaluate the more comprehensive effects of collagen at the cellular level. The lack of statistical significance of the results may also suggest that the effect of collagen alone is not strong enough under cell culture conditions, but may become more pronounced in combination with additional factors such as cellular stress, inflammatory stimuli, or tumor microenvironment. The actual tumor microenvironment consists of dynamic interactions of immune cells, stromal cells, and various matrix components. Thus, given the limitations of in vitro models, it would be meaningful to re-evaluate collagen effects under more complex conditions (using co-culture systems or 3D organoid models).

The significant decrease in CA19-9 levels in HCT116 cells following collagen treatment highlights the potential impact of collagen on tumor marker expression. CA19-9 is commonly used as a biomarker in colorectal cancer, and its reduction may reflect changes in tumor cell activity or viability [31]. However, the lack of significant changes in other tumor markers such as CEA, CA72-4, and CYFRA 21-1 suggests that collagen’s effects may be specific to certain markers or pathways.

Further studies focusing on specific oxidative stress markers, such as reactive oxygen species production, lipid peroxidation, and antioxidant enzyme activities, are warranted to gain deeper insights into the antioxidative mechanisms of double-hydrolyzed collagen peptides.

## 5. Conclusions

In this study, the effects of double-hydrolyzed collagen peptides were evaluated on HCT116 colon carcinoma cells and CCD-18Co normal colon fibroblasts. Treatment with 1 µg/mL collagen significantly decreased cell viability (*p* = 0.0045) and migration (56.86 ± 6.89% vs. 81.31 ± 2.65%, *p* < 0.0001) in HCT116 cancer cells, while promoting fibroblast proliferation (*p* = 0.0143) without inducing cytotoxicity. In dose-dependent viability analysis, the number of viable HCT116 cells was 2.829 (0.5 µg/mL group), 1.605 (1 µg/mL group), and 0.162 (1.5 µg/mL group), indicating the strongest anti-proliferative effect at 1 µL. Interestingly, a slight increase in viability at 1.5 µg/mL suggests a potential biphasic response at higher doses. For CCD-18Co fibroblast cells, the number of viable cells was 1.921 (0.5 µg/mL group), 3.630 (1 µg/mL group), and 3.245 (1.5 µg/mL group), confirming that collagen treatment did not cause cytotoxic effects in normal fibroblasts and selectively targeted malignant cells. Oxidative stress analysis revealed that collagen treatment significantly reduced TOS levels (7.69 ± 1.54 vs. 14.29 ± 0.88 µmol H_2_O_2_ Equiv./L, *p* = 0.0075) and increased TAS levels (0.103 ± 0.077 vs. 0.003 ± 0.006 mmol Trolox Equiv./L, *p* = 0.0095) in HCT116 cells, indicating improved oxidative balance. No significant oxidative changes were observed in CCD-18Co cells. Regarding tumor markers, a significant decrease was detected in CA19-9 levels after collagen treatment (1.04 ± 0.21 vs. 4.61 ± 0.69, *p* = 0.013) in HCT116 cells, while CEA, CA72-4, and CYFRA 21-1 levels remained unchanged. These findings suggest that double-hydrolyzed collagen peptides selectively inhibit proliferation and migration of colon cancer cells, improve antioxidant defenses, and reduce specific tumor marker levels, while having minimal impact on normal fibroblast cells. Although this study presents preliminary data, it highlights the potential therapeutic application of collagen peptides in colorectal cancer management and warrants further detailed mechanistic and in vivo investigations.

## Figures and Tables

**Table 1:** The migration rate (%) of the cell scratch assay.

	Migration Rate of CCD-18Co Cells	Migration Rate of HCT116 Cells
Control	48.54 ± 9.66	81.31 ± 2.65
1 µg/mL Collagen	45.81 ± 5.51	56.86 ± 6.89
*p* value	0.559	*<0.0001*

**Table 2:** Total oxidative status (TOS) and total antioxidant status (TAS) levels in CCD-18Co and HCT116 cell groups treated with 1 µg/mL collagen.

**CCD-18Co Groups**	**TOS (µmol H_2_O_2_ Equiv./L)**	**TAS (mmol Trolox Equiv./L)**
Control	7.273 ± 4.273	0.305 ± 0.207
1 µg/mL Collagen	13.365 ± 10.976	0.040 ± 0.017
*p* value	0.686	0.400
**HCT116 Groups**	**TOS (µmol H_2_O_2_ Equiv./L)**	**TAS (mmol Trolox Equiv./L)**
Control	14.29 ± 0.88	0.003 ± 0.006
1 µg/mL Collagen	7.69 ± 1.54	0.103 ± 0.077
*p* value	*0.0075*	*0.0095*

TOS: total oxidant status, TAS: total antioxidant status.

**Table 3:** Levels of tumor markers CA19-9, CEA, CA72-4, and CYFRA 21-1 in HCT116 cell groups treated with 1 µg/mL collagen.

HCT116 Groups	CA19-9	CEA	CA72-4	CYFRA 21-1
Control	4.61 ± 0.69	<0.2	1.03 ± 0.46	2.71 ± 0.64
1 µg/mL Collagen	1.04 ± 0.21	<0.2	0.84 ± 0.53	2.73 ± 0.32
*p* value	*0.013*	-	0.593	0.857

## Data Availability

The data and materials obtained and analyzed in this study are available from the corresponding author upon reasonable request.

## References

[1] Riegler J., Labyed Y., Rosenzweig S., Javinal V., Castiglioni A., Dominguez C.X., Long J.E., Li Q., Sandoval W., Junttila M.R. (2018). Tumor elastography and its association with collagen and the tumor microenvironment. Clin. Cancer Res..

[2] Le C.C., Bennasroune A., Langlois B., Salesse S., Boulagnon-Rombi C., Morjani H., Dedieu S., Appert-Collin A. (2020). Functional interplay between collagen network and cell behavior within tumor microenvironment in colorectal cancer. Front. Oncol..

[3] Bhattacharjee S., Hamberger F., Ravichandra A., Miller M., Nair A., Affo S., Filliol A., Chin L., Savage T.M., Yin D. (2024). Tumor restriction by type I collagen opposes tumor-promoting effects of cancer-associated fibroblasts. J. Clin. Investig..

[4] Shi R., Zhang Z., Zhu A., Xiong X., Zhang J., Xu J., Sy M.S., Li C. (2022). Targeting type I collagen for cancer treatment. Int. J. Cancer.

[5] Baldari S., Di Modugno F., Nisticò P., Toietta G. (2022). Strategies for efficient targeting of tumor collagen for cancer therapy. Cancers.

[6] Coulson-Thomas V.J., Coulson-Thomas Y.M., Gesteira T.F., de Paula C.A., Mader A.M., Waisberg J., Pinhal M.A., Friedl A., Toma L., Nader H.B. (2011). Colorectal cancer desmoplastic reaction up-regulates collagen synthesis and restricts cancer cell invasion. Cell Tissue Res..

[7] Dibdiakova K., Svec A., Majercikova Z., Adamik M., Grendar M., Vana J., Ferko A., Hatok J. (2022). Associations between matrix metalloproteinase, tissue inhibitor of metalloproteinase and collagen expression levels in the adjacent rectal tissue of colorectal carcinoma patients. Mol. Clin. Oncol..

[8] Mármol I., Sánchez-de-Diego C., Pradilla Dieste A., Cerrada E., Rodriguez Yoldi M.J. (2017). Colorectal carcinoma: A general overview and future perspectives in colorectal cancer. Int. J. Mol. Sci..

[9] Alsheridah N., Akhtar S. (2018). Diet, obesity and colorectal carcinoma risk: Results from a national cancer registry-based middle-eastern study. BMC Cancer.

[10] Astin M., Griffin T., Neal R.D., Rose P., Hamilton W. (2011). The diagnostic value of symptoms for colorectal cancer in primary care: A systematic review. Br. J. Gen. Pract..

[11] Xie Y.H., Chen Y.X., Fang J.Y. (2020). Comprehensive review of targeted therapy for colorectal cancer. Signal Transduct. Target. Ther..

[12] Xu S., Xu H., Wang W., Li S., Li H., Li T., Zhang W., Yu X., Liu L. (2019). The role of collagen in cancer: From bench to bedside. J. Transl. Med..

[13] Necula L., Matei L., Dragu D., Pitica I., Neagu A., Bleotu C., Diaconu C.C., Chivu-Economescu M. (2022). Collagen family as promising biomarkers and therapeutic targets in cancer. Int. J. Mol. Sci..

[14] Karlíková M., Čurillová M., Pecen L., Karnos V., Topolčan O. (2024). Evaluation of serum cytokeratines, thymidine kinase, and growth factors as cancer biomarkers in colorectal cancer. Anticancer Res..

[15] Liska V., Treska V., Skalicky T., Fichtl J., Bruha J., Vycital O., Topolcan O., Palek R., Rosendorf J., Polivka J. (2017). Evaluation of tumor markers and their impact on prognosis in gallbladder, bile duct and cholangiocellular carcinomas–A pilot study. Anticancer Res..

[16] Dogan R., Guler E.M., Kocyigit A., Çelik I., Senturk E., Yenigun A., Tugrul S., Ozturan O. (2021). Are the oxidative stress levels in the tumor center and tumor boundary different from those in healthy tissue?. Eur. Arch. Oto Rhino Laryngol..

[17] Republic of Türkiye, Ministry of Agriculture and Forestry List of Restricted Substances for Food Supplements. https://www.tarimorman.gov.tr/GKGM/Belgeler/DB_Gida_Isletmeleri/Takviye_Edici_Gidalar_Kisitli_Maddeler_Listesi.pdf.

[18] Inacio P.A.Q., Chaluppe F.A., Aguiar G.F., Coelho C.F., Vieira R.P. (2024). Effects of hydrolyzed collagen as a dietary supplement on fibroblast activation: A systematic review. Nutrients.

[19] Song J., Sun D.L., Li C.Y. (2024). TL1A promotes fibrogenesis in colonic fibroblasts via the TGF-β1/Smad3 signaling pathway. Curr. Med. Sci..

[20] Koh B., Jeon H., Kim D., Kang D., Kim K.R. (2019). Effect of fibroblast co-culture on the proliferation, viability and drug response of colon cancer cells. Oncol. Lett..

[21] Yu Y., Liu D., Liu Z., Li S., Ge Y., Sun W., Liu B. (2018). The inhibitory effects of COL1A2 on colorectal cancer cell proliferation, migration, and invasion. J. Cancer.

[22] Addis R., Cruciani S., Santaniello S., Bellu E., Sarais G., Ventura C., Maioli M., Pintore G. (2020). Fibroblast proliferation and migration in wound healing by phytochemicals: Evidence for a novel synergic outcome. Int. J. Med. Sci..

[23] Nandhini J., Karthikeyan E., Sheela M., Bellarmin M., Kannan B.G., Pavithra A., Sri D.S., Prakash S.S., Kumar S.R. (2025). Optimization of microwave-assisted green synthesis of zinc oxide nanoparticles using Ocimum americanum and Euphorbia hirta extracts: In vitro evaluation of antioxidant, anti-inflammatory, antibacterial, cytotoxicity, and wound healing properties. Intell. Pharm..

[24] Gabr S.A., Elsaed W.M., Eladl M.A., El-Sherbiny M., Ebrahim H.A., Asseri S.M., Eltahir Y.A., Elsherbiny N., Eldesoqui M. (2022). Curcumin modulates oxidative stress, fibrosis, and apoptosis in drug-resistant cancer cell lines. Life.

[25] Fang M., Yuan J., Peng C., Li Y. (2014). Collagen as a double-edged sword in tumor progression. Tumor Biol..

[26] Zou X., Feng B., Dong T., Yan G., Tan B., Shen H., Huang A., Zhang X., Zhang M., Yang P. (2013). Up-regulation of type I collagen during tumorigenesis of colorectal cancer revealed by quantitative proteomic analysis. J. Proteom..

[27] Wei W., Wang J., Huang P. (2023). Tumor necrosis factor-α induces proliferation and reduces apoptosis of colorectal cancer cells through STAT3 activation. Immunogenetics.

[28] Chen Q., Chen O., Martins I.M., Hou H., Zhao X., Blumberg J.B., Li B. (2017). Collagen peptides ameliorate intestinal epithelial barrier dysfunction in immunostimulatory Caco-2 cell monolayers via enhancing tight junctions. Food Funct..

[29] Bruzzone C.M., Belcher J.D., Schuld N.J., Newman K.A., Vineyard J., Nguyen J., Chen C., Beckman J.D., Steer C.J., Vercellotti G.M. (2008). Quantitative real-time polymerase chain reaction (qRT-PCR) restriction fragment length polymorphism (RFLP) method for monitoring highly conserved transgene expression during gene therapy. Transl. Res..

[30] Liu X., Shi F., Li Y., Yu X., Peng S., Li W., Luo X., Cao Y. (2016). Post-translational modifications as key regulators of TNF-induced necroptosis. Cell Death Dis..

[31] Singh S., Kumar R., Kumar U., Kumari R. (2020). Clinical significance and role of TK1, CEA, CA 19-9 and CA 72-4 levels in diagnosis of colorectal cancers. Asian Pac. J. Cancer Prev..

